# File-Specific Cyclic Fatigue Resistance of NiTi Instruments After Repeated Use in Simulated Canals: Patterns Compatible with Potential Stress-Induced Martensite Transformation Effects

**DOI:** 10.3390/ma19050866

**Published:** 2026-02-26

**Authors:** Hyeonu Jo, Sang Won Kwak, Jung-Hong Ha, Asgeir Sigurdsson, Hyeon-Cheol Kim

**Affiliations:** 1 Department of Conservative Dentistry, School of Dentistry, Dental Research Institute, Dental and Life Science Institute, Pusan National University, Yangsan 50612, Republic of Korea; hy5959546@gmail.com (H.J.); endokwak@pusan.ac.kr (S.W.K.); 2 Department of Conservative Dentistry, School of Dentistry, Kyungpook National University, Daegu 41566, Republic of Korea; endoking@knu.ac.kr; 3 Department of Endodontics, New York University College of Dentistry, New York, NY 10010, USA; asgeir.sigurdsson@nyu.edu

**Keywords:** cyclic fatigue, file sequences, fracture risk, number of cycles to fracture, reuse, SIM transformation

## Abstract

This study evaluated changes in the number of cycles to fracture (NCF) of Nickel–Titanium (NiTi) files after repeated use in simulated canals and investigated the potential relationship with stress-induced martensite (SIM) transformation effect. A total of 225 ProTaper Ultimate (PTUL) files were divided into three groups: Group 1 consisted of new files, Group 2 comprised files used to shape two resin simulated canals, and Group 3 consisted of files used to shape four canals. The simulated resin canals with a 16 mm length of J-shaped with 35° curvature were prepared using PTUL Slider, Shaper, F1, F2, and F3 files sequentially. After instrumentation, the cyclic fatigue resistance of each sequential file was assessed in a 35° curved steel canal by rotating at 400 rpm using a custom-made device. Statistical analysis was performed using one-way ANOVA with Tukey’s post hoc test or Kruskal–Wallis with Dunn’s test with Bonferroni correction for parametric and non-parametric data, respectively. Slider and Shaper maintained stable NCF across all groups (*p* > 0.05). In contrast, F1 showed a transient increase (117.7%) after two uses but declined significantly (91.6%) after four uses (*p* < 0.05). F2 and F3 demonstrated progressive NCF reductions (F2: 72.9%; F3: 71.5% after four uses), with F3 showing the most pronounced decline (*p* < 0.05). Repeated use of NiTi files reduced their cyclic fatigue resistance in a file-specific manner, with larger finishing files most affected. The distinctive F1 pattern suggests potential preload-related or SIM transformation effects that warrant further metallurgical investigation.

## 1. Introduction

Nickel–Titanium (NiTi) alloys are widely used in endodontic file systems because of their unique properties, such as superelasticity and shape memory [1]. These properties allow NiTi files to navigate complex and curved root canal anatomies with greater ease compared to traditional stainless-steel files [2]. The flexibility and durability of NiTi files reduce the risk of procedural errors such as ledge formation, transportation, and perforation, making them essential tools for effective root canal shaping [3]. However, despite these advantageous features, NiTi files are susceptible to unexpected fracture within the root canal, particularly under cyclic fatigue loading [4].

Cyclic fatigue occurs when a rotating file is subjected to repeated tensile and compressive stresses in a curved canal, leading to the initiation and propagation of micro-cracks within the file structure and eventually causing fracture without visible plastic deformation [5,6,7]. The resistance of a file to cyclic fatigue is therefore crucial, because it directly affects the instrument’s durability and its ability to perform safe shaping during root canal treatments [5,8].

The cyclic fatigue resistance of NiTi instruments is influenced by several factors, including alloy composition, thermomechanical treatment, instrument design (taper, cross-sectional geometry, and core diameter), and operating conditions such as canal curvature, rotational speed, and temperature [5,8]. Heat-treated NiTi alloys (e.g., M-Wire, Gold-wire, Blue-wire) have been developed to enhance flexibility and fatigue resistance compared with conventional austenitic NiTi, although often at the expense of torsional strength [9]. Recent studies have highlighted that temperature-dependent phase transformations between austenite, R-phase, and martensite can substantially alter the mechanical behavior and fatigue life of NiTi instruments under clinical conditions [9,10,11].

During root canal preparation, instruments experience combined cyclic fatigue and/or torsional stresses, and these loads become particularly relevant when files are used repetitively [9,10]. Depending on the file size and geometry, the preloaded stress during the usage may result in different levels of stress-induced martensite (SIM) transformation, which may influence the fatigue resistance of the files. SIM transformation is a key phenomenon in NiTi alloys whereby mechanical loading promotes the conversion of austenite (or R-phase) to martensite [9,11,12]. Experimental studies in NiTi wires and endodontic instruments have suggested that torsional preloading can change subsequent cyclic fatigue resistance, and this effect has been attributed, at least in part, to SIM and associated microstructural rearrangements [10]. However, direct metallurgical evidence linking SIM to changes in fatigue behavior of clinically used rotary files remains limited, and most available data are indirect, based on mechanical performance rather than detailed phase or microstructural analysis.

Manufacturers have continuously developed NiTi files with diverse designs and material properties to meet the evolving demands of endodontic treatments [5,7,11]. Advances in design have introduced variations in cross-sectional geometry and taper, optimizing file performance across different canal anatomies [10]. Additionally, heat treatment processes have been employed to modify the metallurgical properties of NiTi alloys, improving their flexibility and resistance to cyclic fatigue [11,12]. These combined advancements reflect the industry’s commitment to creating durable and reliable instruments in clinical practice [13].

Among the various NiTi file systems, the ProTaper Ultimate (PTUL; Dentsply Sirona, Ballaigues, Switzerland) represents an advanced evolution of previous systems such as ProTaper Gold (Dentsply Sirona) and ProTaper Next (Dentsply Sirona) [14]. Each file in the PTUL system undergoes a specific heat treatment process engineered to enhance its performance, providing different levels of flexibility and cyclic fatigue resistance [15]. The sequence includes five specific files, incorporating an M-Wire Slider and Gold-wire shaping (Shaper) and finishing files (F1, F2, and F3), each with distinct geometries and thermal properties engineered for its specific role in the sequence [16].

Previous investigations have evaluated the shaping ability, canal preservation, and mechanical properties of PTUL and related systems, but there is still limited information on how repeated use of each file within the sequence affects cyclic fatigue resistance under standardized conditions, and whether any observed changes might be compatible with SIM-related effects [17,18]. The rate of fatigue degradation may vary substantially depending on the file design and specific heat treatment. In the PTUL system, these factors are especially crucial, as each file is uniquely engineered with distinct heat treatment properties that influence its performance under cyclic stress.

In clinical practice, NiTi rotary instruments are often reused despite manufacturer recommendations for single use, especially in regions where economic or logistical factors encourage reuse after cleaning and sterilization. Repeated use may alter the fatigue behavior of instruments in a file sequence in different ways depending on their size, cross-sectional metal volume, and alloy/heat treatment. Understanding these file-specific changes is essential for developing safer, evidence-based reuse protocols, for identifying which instruments within a sequence are most vulnerable to fatigue-related fracture, and for informing future instrument and sequence design.

Therefore, the primary aim of this study was to evaluate how repeated use affects the cyclic fatigue resistance of each ProTaper Ultimate file (Slider, Shaper, F1, F2, F3) under standardized conditions in simulated canals. The null hypothesis was that there would be no significant difference in the reduction in the number of cycles to fracture (NCF) among files according to the number of canals instrumented. In addition, as a working, exploratory hypothesis, it was considered that any changes in NCF with repeated use might differ among file types in a pattern that could be compatible with SIM-related effects, particularly in heat-treated instruments. This mechanistic hypothesis is presented as indirect and hypothesis-generating only, because no direct metallurgical analyses of phase transformation were performed in the present study.

## 2. Materials and Methods

**Figure 1:**
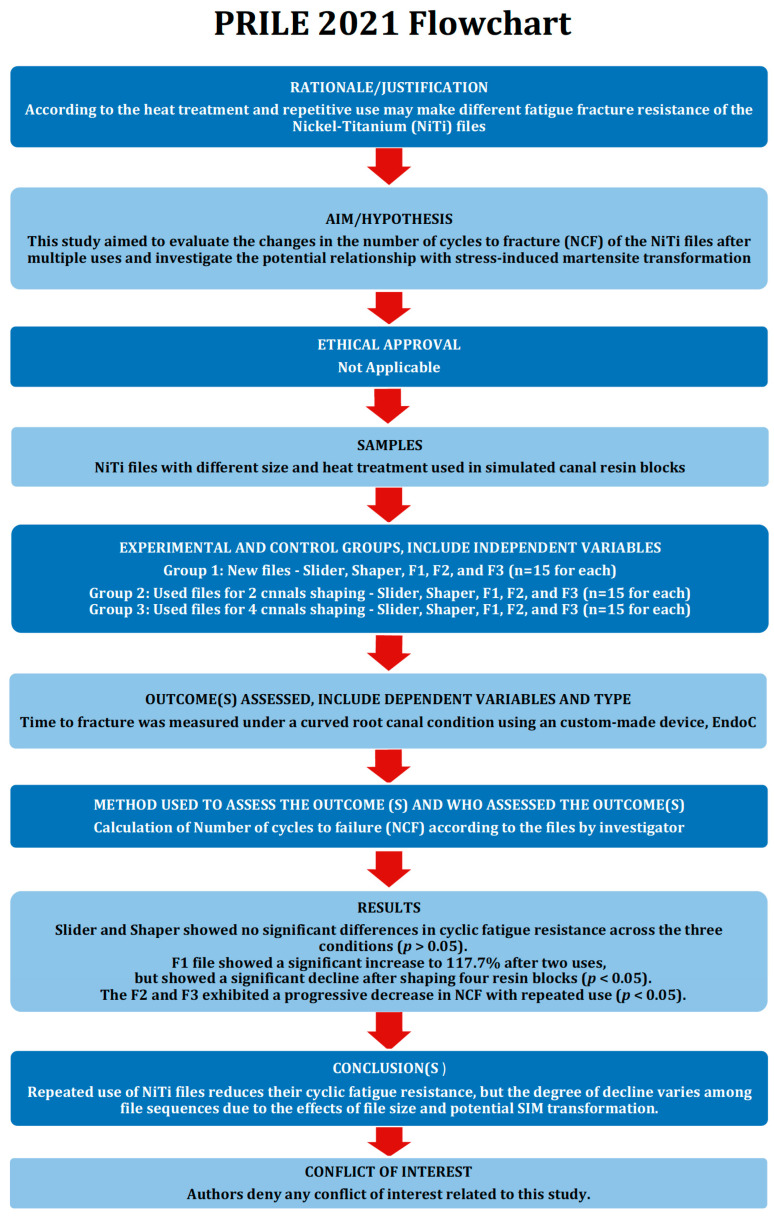
The PRILE 2021 flowchart.

The manuscript of this laboratory study was prepared and reported according to the Preferred Reporting Items for Laboratory studies in Endodontology (PRILE) 2021 guidelines (Figure 1).

The ProTaper Ultimate (PTUL; Dentsply Sirona, Ballaigues, Switzerland) rotary instruments were used, comprising five file types: Slider, Shaper, F1, F2, and F3.

The Slider is a small-taper orifice and glide-path instrument with a relatively slim core and convex triangular cross-section, designed to shape the coronal and middle thirds with high flexibility. According to the manufacturer, the Slider is manufactured from M-Wire alloy and exhibits austenite/R-phase-based superelastic behavior, whereas the Shaper and finishing files (F1–F3) are produced from Gold-wire alloy and present predominantly martensitic behavior with enhanced flexibility and cyclic fatigue resistance. The Shaper has a progressively tapered design and modified triangular cross-section, intended to enlarge the coronal and middle thirds while preserving canal curvature. The finishing files F1, F2, and F3 have increasing tip sizes and fixed tapers, each presenting an off-centered or modified triangular cross-section and larger core diameter than the preceding file, to complete the apical and final shaping. All instruments have a 1.1 mm maximum shank diameter, consistent with the minimally invasive design concept of the PTUL system.

### 2.1. Group Designation and Mechanical Loading

A total of 225 PTUL rotary files were used in this study and allocated into three experimental groups to investigate the effects of repeated use on cyclic fatigue resistance: Group 1 (*n* = 75), new and unused files (baseline); Group 2 (*n* = 75), files used to prepare two simulated canals; and Group 3 (*n* = 75), files used to prepare four simulated canals.

Each group comprised 15 files of each type (Slider, Shaper, F1, F2, and F3). The sample size (*n* = 15 per file type per group) was determined based on both precedent in similar cyclic fatigue studies and an a priori power estimation. Previous research evaluating cyclic fatigue resistance of NiTi rotary instruments has commonly used 10–15 specimens per experimental group. In addition, an a priori power analysis (α = 0.05; power = 0.80) based on effect sizes and variance values derived from preliminary pilot measurements and previously published NCF data for comparable instruments indicated that a minimum of 13 specimens per subgroup would be required to detect a statistically significant difference in NCF among usage conditions. Therefore, 15 files per subgroup were to ensure adequate statistical power and to compensate for potential variability [19].

Simulated canals were prepared in standardized resin blocks (Endo Training Bloc; Dentsply Sirona) containing a single J-shaped canal of 16 mm length, with a curvature of 35°, as specified by the manufacturer. Standardized resin blocks were selected to ensure identical canal length, curvature, and radius across all specimens, thereby minimizing variability in mechanical loading conditions during instrumentation. This approach allowed the effect of repeated use on cyclic fatigue resistance to be isolated from anatomical variability inherent to natural teeth.

The shaping procedure began with the establishment of canal patency using a #10 K-file (Mani, Inc., Tochigi, Japan). Once patency was achieved, the PTUL files were used sequentially, starting with the Slider, followed by the Shaper, F1, F2, and finally F3. All preparations were performed by a single postgraduate endodontic resident with 4 years of clinical experience in rotary instrumentation to reduce operator variability.

Before the experimental phase, the operator performed a series of calibration procedures using additional resin blocks to standardize the 3 mm pecking motion, working length control, irrigation protocol, and debris removal technique. These preliminary procedures were conducted to ensure consistent instrumentation dynamics across all specimens.

Each NiTi file was used with a gentle 3 mm pecking motion to the working length. After every three pecking strokes, the file was withdrawn for canal irrigation the canal with saline solution and removal of resin debris from the flutes. This cycle was repeated until a total of six pecking strokes were completed and the file reached the full working length of 16 mm. Files were then removed, cleaned, and assigned to the subsequent cyclic fatigue test according to their designated group.

### 2.2. Cyclic Fatigue Test

**Figure 2:**
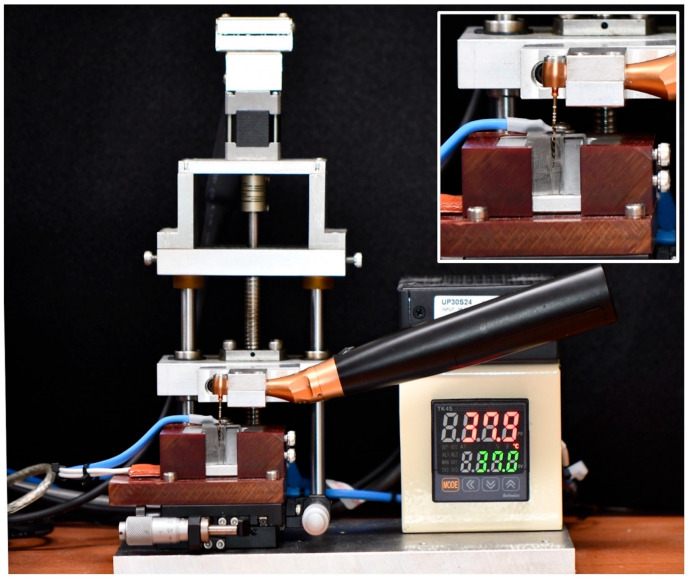
Experimental set-up using a custom-made device (EndoC; DMJ systems, Busan, Republic of Korea) with an endodontic motor (Ai-Motor, Guilin Woodpecker Medical Instrument Co., Ltd., Guilin, China) and an electric heat controller for body temperature (37 °C).

After the simulated shaping procedures, cyclic fatigue tests were performed on all files using a custom-made device (EndoC; DMJ System, Busan, Republic of Korea), designed to simulate the rotational motion within a curved canal environment (Figure 2). According to the method of Schnieder, an artificial canal model made of tempered steel was used, with a 0.6 mm apical diameter, a 35° curvature, and a 6.06 mm radius [20].

Each file was mounted in an endodontic motor (Ai-Motor; Guilin Woodpecker Medical Instrument Co., Ltd., Guilin, China) attached to the EndoC device. The files were rotated at 400 rpm with a maximum torque of 4.0 N·cm. The rotational speed and torque settings were selected in accordance with the manufacturer’s Instructions for Use for ProTaper Ultimate instruments, which recommend continuous rotation at 400 rpm with torque values within the range of 4.0–5.2 N·cm. A torque setting of 4.0 N·cm was chosen as a standardized and conservative value within this recommended range to reflect common clinical practice while minimizing excessive torsional overload during testing.

The device produced a continuous dynamic up-and-down pecking movement (4 mm in each direction at 0.5-s intervals in both directions). Before each testing session, the alignment of the artificial canal, rotational parameters, and temperature control system were verified to ensure consistent experimental conditions. Pilot measurements confirmed stable and reproducible NCF values under identical settings.

All tests were performed at body temperature (37 °C), which was controlled by an electronic heat controller (TK4N/S/SP Autonics, Busan, Republic of Korea) and monitored using an external thermocouple in contact with the metallic canal block. Maintaining body temperature was considered essential because the mechanical behavior and phase composition of heat-treated NiTi alloys are temperature-dependent. Each file was rotated until fracture occurred, at which point the time to fracture (seconds) was recorded with a digital chronometer. The number of cycles to failure (NCF) for each file was calculated by multiplying the recorded time (seconds) by the rotational speed (rpm).

### 2.3. Statistical Analysis

NCF values were recorded for each file and group. All statistical analyses were performed using commercially available statistical software (SPSS version 25.0; IBM Corp., Armonk, NY, USA). The normality of the data distribution for each file type was assessed using the Shapiro–Wilk test (*p* > 0.05 indicating normal distribution), and homogeneity of variances among groups was evaluated using Levene’s test.

Based on these assumptions, different inferential tests were applied with a significance level of α = 0.05. For file types in which both normality and homogeneity were satisfied (Slider, Shaper, F2), one-way analysis of variance (ANOVA) was used to compare NCF among the three usage groups, followed by Tukey’s post hoc test for pairwise comparisons. For file types in which normality or homogeneity assumptions were violated (F1 and F3), the Kruskal–Wallis test was used to compare groups, followed by Dunn’s post hoc test with Bonferroni correction for multiple comparisons.

All statistical analyses were performed separately for each file type to evaluate file-specific changes in cyclic fatigue resistance according to the number of simulated canals prepared. Effect sizes were also calculated where appropriate to aid interpretation of the magnitude of differences among groups. A *p*-value < 0.05 was considered statistically significant.

## 3. Results

**Figure 3:**
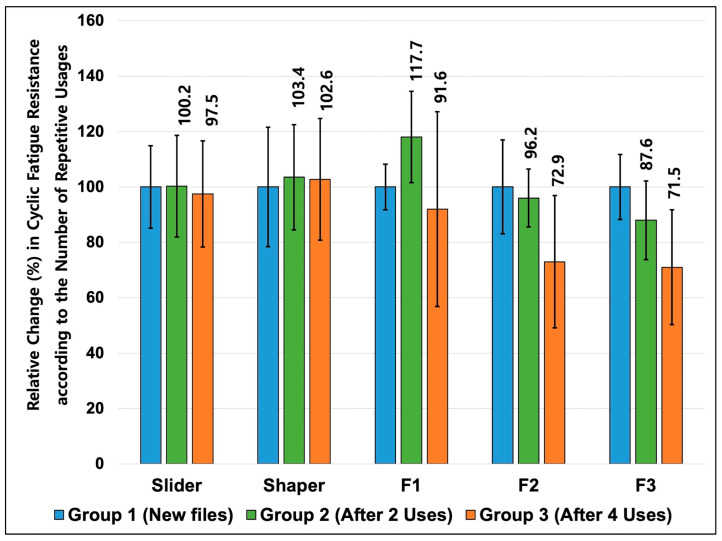
Relative changes (reduction or increase, %) in cyclic fatigue resistance of the sequential ProTaper Ultimate files according to the number of repetitive usages. The values for Groups 2 and 3 are expressed as percentages of the corresponding new files in Group 1 (100%).

The NCF values for each file type under different usage conditions are presented in Table 1. Figure 3 illustrates the relative changes in NCF according to the number of canals instrumented, with the values for new files (Group 1) set as 100%. Overall, repeated use produced file-specific patterns of change in cyclic fatigue resistance.

The Slider and Shaper files showed no statistically significant differences in cyclic fatigue resistance among the three conditions (*p* > 0.05). The NCF of the Slider remained close to the baseline value, corresponding to 100.2% and 97.5% of the new-file value after two and four uses, respectively. The Shaper file exhibited a similar tendency, with NCF ratios of 103.4% after two uses and 102.6% after four uses compared with the new file. These findings indicate a relatively stable fatigue behavior across repeated uses for these smaller instruments.

In contrast, the F1 file showed a statistically significant increase in NCF to 117.7% after two uses, followed by a significant decrease after shaping four resin block canals (*p* < 0.05). After four uses, the NCF of F1 decreased to 91.6% of the baseline value. This pattern reflects a non-linear response to repeated use, characterized by an initial increase followed by subsequent reduction.

The F2 and F3 files exhibited a progressive reduction in NCF with repeated use (*p* < 0.05). For F2, the NCF decreased to 96.2% after two uses and to 72.9% after four uses. For F3, the NCF declined to 87.6% after two uses and to 71.5% after four uses, with significant differences among all three usage conditions (*p* < 0.05). Among the evaluated instruments, the F3 file showed the most pronounced and consistent reduction in cyclic fatigue resistance as the number of uses increased.

Taken together, these results demonstrate that the effect of repeated use on cyclic fatigue resistance was dependent on file type and size within the PTUL sequence.

## 4. Discussion

This study evaluated the effect of repeated use on the cyclic fatigue resistance of each ProTaper Ultimate (PTUL) file under standardized conditions. The null hypothesis—that there would be no significant difference in NCF reduction among file types according to the number of canals instrumented—was rejected.

The results revealed file-specific patterns: the Slider and Shaper maintained stable NCF values across all usage conditions, while F2 and F3 exhibited progressive declines in fatigue resistance. Notably, F1 showed a transient increase in NCF after two uses followed by a subsequent decrease, highlighting non-linear fatigue behavior within the same heat-treated alloy system.

The PTUL system incorporates distinct alloys and geometries within a single sequence, featuring a slender 1.1 mm shank diameter optimized for minimally invasive endodontics [16,21]. The Slider (M-Wire, austenitic/R-phase, slim core, convex triangular cross-section) and Shaper (Gold-wire, progressively tapered, modified triangular cross-section) demonstrated remarkable stability, with NCF values remaining close to 100% of baseline after four uses. This stability is consistent with previous reports indicating that instruments with smaller core diameters and reduced cross-sectional metal mass exhibit higher baseline cyclic fatigue resistance and less degradation during repeated use.

Despite the Slider’s superelastic behavior (Af temperature above body temperature), it achieved the highest NCF among tested files [2,22,23]. This finding suggests that geometric factors, including core diameter and cross-sectional metal mass, may exert a stronger influence on cyclic fatigue behavior than phase constitution alone under the present experimental conditions. The Slider file exhibits an austenite finish (Af) temperature above body temperature, and its martensite peak temperature lies below body temperature, thus allowing the instrument to exhibit superelastic behavior under clinical conditions [16,24]. Although superelastic instruments are generally considered to exhibit lower cyclic fatigue resistance compared with heat-treated martensitic-dominant files, the present findings suggest that geometric factors, such as reduced core diameter and metal mass, may exert a stronger influence on fatigue behavior than phase constitution alone under the tested conditions. Furthermore, the absence of significant NCF reduction after repeated use indicates that smaller instruments may accumulate fatigue damage more slowly than larger finishing files within the same system [19,25].

The Shaper file also maintained stable fatigue resistance across repeated uses. Although slight increases in NCF were observed, these differences were not statistically significant. This consistent behavior may reflect the combined effects of Gold-wire heat treatment and moderate cross-sectional geometry; however, the present study was not designed to isolate metallurgical contributions from geometric influences [23].

The F1 file, also made of Gold-wire, exhibited a different pattern of NCF changes with repeated use. This transient and unusual improvement may result from torsional pre-loading during canal shaping, which could enhance cyclic fatigue resistance through stress-induced martensite (SIM) transformation [9,10]. During the initial uses, torsional preloading could have promoted a partial transformation of the alloy from austenite to martensite, thereby temporarily enhancing the cyclic fatigue resistance. However, with repeated use, the accumulation of microstructural defects and microcracks likely counteracted this benefit. After four uses, the NCF decreased to 91.6% of the baseline, although the difference between Groups 1 and 3 was not significant. While the F1 file experienced a certain amount of torsional loading during canal preparation to induce SIM transformation, the Slider and Shaper may not have reached the stress threshold required to trigger SIM transformation, which could explain the absence of significant changes in their fatigue resistance. However, because this study included no metallurgical analyses (DSC, XRD, SEM/TEM) or fractographic examination, SIM remains a hypothesis-generating interpretation rather than a confirmed mechanism. Alternative explanations—including residual stress redistribution, subtle geometric changes from debris removal, or statistical variation—cannot be excluded and warrant further investigation through integrated mechanical-metallurgical approaches

In contrast, the larger finishing files F2 and F3 (Gold-wire, larger core diameters) showed marked NCF reductions after four uses (F2: 72.9%, F3: 71.5%). These findings indicate that, for instruments with larger core diameters and greater metal mass, cumulative cyclic fatigue damage predominates over any potential preload-related strengthening effects. The larger diameters or metal volumes of these files may have naturally reduced their flexibility, thereby diminishing the benefits of torsional pre-loading. Consequently, the F3 file showed a more pronounced decrease in NCF than either F1 or F2 files. From a clinical perspective, these findings have practical implications. In multicanal teeth, a single file sequence is often used across several canals during one treatment session, effectively constituting repeated use. The present results suggest that larger finishing files (particularly F3, and to a lesser extent F2) may be more vulnerable to fatigue-related fracture after repeated use, whereas smaller instruments such as the Slider and Shaper demonstrated greater resistance to fatigue degradation under standardized conditions. Accordingly, reuse protocols may need to be more conservative for larger finishing instruments, especially in curved canals.

Manufacturers typically recommend single-use of files to minimize the risk of unexpected intracanal fracture. However, in clinical practice—particularly in multicanal teeth such as molars—the same file sequence is frequently used across multiple canals within a single treatment session, effectively constituting repeated use. Even this limited reuse may increase cumulative fatigue damage and fracture risk, necessitating careful clinical judgment [26]. Understanding how cyclic fatigue changes with repeated use could help clinicians limit file reuse and mitigate fracture risks. The present findings suggest that the Slider and Shaper files exhibit minimal changes in NCF even with multiple uses, whereas files with larger diameters (F2, F3) show substantial reductions in NCF. Despite the cyclic fatigue benefits conferred by heat treatment, the reduction in NCF was more pronounced in larger diameter files than smaller instruments. This indicates that—within this system—instrument diameter and cross-sectional metal volume exert a strong influence on repeated-use fatigue behavior that may outweigh heat treatment advantages under the tested conditions [27].

This study was conducted using artificial resin blocks with standardized canal shapes and curvatures to give constant stable loading conditions during simulated instrumentation. While this approach provides reproducibility, it does not fully reflect the variability and complexity of natural root canals. Additionally, the rotational force and pecking depth applied during the cyclic fatigue test may not fully represent the dynamic forces experienced in clinical settings. Although smaller instruments, such as the Slider and Shaper, showed no statistically significant reduction in cyclic fatigue under the present conditions, this should not be interpreted as evidence of clinical safety with repeated reuse, as the absence of significant changes may simply reflect insufficient fatigue accumulation; therefore, further investigations are required to clarify their behavior under more clinically relevant scenarios.

SIM transformation in NiTi alloys occurs when mechanical stress promotes the conversion of the austenitic phase into martensite [10,12]. This transformation can alter the mechanical behavior of the alloy and influence its fatigue resistance [9]. Previous studies have demonstrated that torsional preloading can alter cyclic fatigue resistance, and such effects have been attributed to SIM transformation [9,10]. However, because the present study relied exclusively on mechanical testing without direct phase or microstructural analysis, any linkage between the observed F1 pattern and SIM remains theoretical. Furthermore, since the phase transformation behavior and mechanical performance of NiTi files are influenced by the type of heat treatment applied during manufacturing, the potential interaction between SIM transformation and heat treatment should be considered when interpreting the fatigue behavior of different file types.

Despite the limitations, this study provides novel, sequence-level data on file-specific fatigue behavior within the PTUL sequence. To our knowledge, it is among the first to systematically compare all five files after identical repeated-use conditions. The identification of size-dependent fatigue degradation within a single system may assist both clinicians in establishing file-specific reuse policies and manufacturers in optimizing sequence design to enhance safety margins.

Future studies are required to investigate the behavior of PTUL files in extracted human teeth with varying canal morphologies and curvatures for better reflect clinical scenarios. Furthermore, the influence of other factors such as torsional fatigue, sterilization processes, and prolonged use on file performance should be explored. In particular, combined mechanical–metallurgical approaches will be required to determine whether SIM-related phenomena meaningfully contribute to the non-linear fatigue responses observed in certain file types. Although the present study did not provide direct evidence of stress-induced martensitic transformation, future investigations employing high-magnification microscopic or metallurgical analyses are expected to clarify its occurrence and role in file performance. Comparative studies involving other commercially available file systems could also provide a broader perspective on the clinical utility and limitations of PTUL files.

## 5. Conclusions

Repeated use of NiTi files reduced the cyclic fatigue resistance of PTUL files in a size- and sequence-dependent manner. Smaller instruments (Slider, Shaper) maintained stable NCF after four uses, while larger finishing files (particularly F3) exhibited marked and progressive reductions in fatigue resistance. The transient increase in NCF observed in F1 after two uses represents a non-linear fatigue response that may be associated with torsional preloading; however, any potential involvement of stress-induced martensitic (SIM) transformation remains speculative in the absence of direct metallurgical analysis.

Within the limitations of this standardized laboratory model using simulated curved resin canals, repeated use of smaller PTUL instruments did not result in significant fatigue degradation, whereas larger finishing files demonstrated clinically relevant reductions in cyclic fatigue resistance. Accordingly, reuse strategies should be tailored according to instrument size and sequence position, and more conservative single-use policies may be advisable for larger finishing files, particularly under complex or highly curved canal conditions.

## Figures and Tables

**Table 1:** Number of cycles to failure (NCF) of each ProTaper Ultimate file according to the number of uses (mean ± standard deviation).

File	Numbers of Usage		
	0 Canals (New One)	2 Canals	4 Canals
Slider	5087 ± 838	5101 ± 935	4961 ± 950
Shaper	1297 ± 256	1341 ± 253	1331 ± 264
F1	1279 ± 105 ^a^	1506 ± 178 ^b^	1171 ± 348 ^a^
F2	1169 ± 193 ^a^	1125 ± 104 ^a^	853 ± 204 ^b^
F3	751 ± 89 ^a^	658 ± 108 ^b^	537 ± 148 ^c^

^a, b, c^: Different superscript letters indicate significant differences between groups in the same row (*p* < 0.05).

## Data Availability

The raw data supporting the conclusions of this article will be made available by the authors on request.

## Abbreviations

NCF	the number of cycles to fracture
NiTi	nickel–titanium
PTUL	ProTaper Ultimate
SIM	stress-induced martensite
